# Dealing with paralogy in RADseq data: in silico detection and single nucleotide polymorphism validation in *Robinia pseudoacacia* L.

**DOI:** 10.1002/ece3.2466

**Published:** 2016-09-22

**Authors:** Cindy F. Verdu, Erwan Guichoux, Samuel Quevauvillers, Olivier De Thier, Yec'han Laizet, Adline Delcamp, Frédéric Gévaudant, Arnaud Monty, Annabel J. Porté, Philippe Lejeune, Ludivine Lassois, Stéphanie Mariette

**Affiliations:** ^1^ Forest Management Unit Gembloux Agro‐Bio Tech University of Liège Gembloux Belgium; ^2^ Biogeco INRA University of Bordeaux Cestas France; ^3^ BFP INRA University of Bordeaux Villenave d'Ornon France; ^4^ Biodiversity and Landscape Unit Gembloux Agro‐Bio Tech University of Liège Gembloux Belgium

**Keywords:** black locust, depth of coverage, putative paralogy filtering, restriction site‐associated DNA sequencing

## Abstract

The RADseq technology allows researchers to efficiently develop thousands of polymorphic loci across multiple individuals with little or no prior information on the genome. However, many questions remain about the biases inherent to this technology. Notably, sequence misalignments arising from paralogy may affect the development of single nucleotide polymorphism (SNP) markers and the estimation of genetic diversity. We evaluated the impact of putative paralog loci on genetic diversity estimation during the development of SNPs from a RADseq dataset for the nonmodel tree species *Robinia pseudoacacia* L. We sequenced nine genotypes and analyzed the frequency of putative paralogous RAD loci as a function of both the depth of coverage and the mismatch threshold allowed between loci. Putative paralogy was detected in a very variable number of loci, from 1% to more than 20%, with the depth of coverage having a major influence on the result. Putative paralogy artificially increased the observed degree of polymorphism and resulting estimates of diversity. The choice of the depth of coverage also affected diversity estimation and SNP validation: A low threshold decreased the chances of detecting minor alleles while a high threshold increased allelic dropout. SNP validation was better for the low threshold (4×) than for the high threshold (18×) we tested. Using the strategy developed here, we were able to validate more than 80% of the SNPs tested by means of individual genotyping, resulting in a readily usable set of 330 SNPs, suitable for use in population genetics applications.

## 1 Introduction

With the extensive development of next‐generation sequencing (NGS) technologies and the accurate bioinformatics treatment of data, it is now feasible to obtain genomic data and develop single nucleotide polymorphism (SNP) markers for nonmodel species (Etter et al., 2011). RADseq is one of the NGS technologies increasingly used for population genetics, phylogeography, SNP development and linkage map construction studies (reviewed by Davey et al., 2011). This method, based on a DNA restriction approach, greatly decreases the proportion of the genome targeted by sequencing (about 0.1%), so as to increase the coverage of sequencing fragments and to ensure accurate genotyping (Davey et al., 2011). The proportion of the genome sequenced may be very small, but the number of markers generated remains very high (several thousands), considerably greater than the number of markers generated by traditional technologies, such as amplified fragment length polymorphism or microsatellites (Davey et al., 2013). This method has already been successfully applied to many species, with and without published genome sequences (Boehm, Waldman, Robinson, & Hickerson, 2015; Sun et al., 2015), and with complex genomes, such as sunflower (Pegadaraju, Nipper, Hulke, Qi, & Schultz, 2013) and cedar (Karam, Lefevre, Dagher‐Kharrat, Pinosio, & Vendramin, 2015).

However, many studies have also shown that NGS data may include errors likely to result in incorrect biological conclusions, such as an artificial excess of homozygotes, false departure from Hardy–Weinberg equilibrium, an overestimation of inbreeding, unreliable inferences about population structure, and incorrect inferences concerning demographic expansion (reviewed by Andrews et al., 2016). Several potential sources of bias concern the RADseq technology. First, the presence of polymorphism in restriction sites generates null alleles, creating false homozygotes which strongly affect diversity estimates and population genetic inferences (Arnold, Corbett‐Detig, Hartl, & Bomblies, 2013; Davey et al., 2013; Gautier et al., 2013; Ilut, Nydam, & Hare, 2014). Second, extensive sequence polymorphism and high GC content may decrease the coverage of sequences, and hence the opportunity to sample each allele for a locus, creating false homozygotes, missing data and inaccurate genotype calling (Davey et al., 2013). Third, sequencing errors can represent an important source of bias in RADseq analyses, which typically admit error rates of 0.1%–1.5%, compared to 0.001% for traditional Sanger sequencing (Mastretta‐Yanes et al., 2015; Shendure & Ji, 2008). High error rates in sequence reads commonly lead to discarding half of the original RADseq data, unless one can refer to a reference genome for comparison (Peterson, Weber, Kay, Fisher, & Hoekstra, 2012). A simple and commonly used method to discard sequencing errors consists in the elimination of singleton loci, that is, SNPs present in only one genotype in a population (Roesti, Salzburger, & Berner, 2012). In addition, many recent studies have stressed the risks associated with the use of a too low minimum depth of coverage (Brockman et al., 2008; Kim et al., 2011; Nielsen, Korneliussen, Albrechtsen, Li, & Wang, 2012). True heterozygotes may be confounded with sequencing errors at low depth (Kim et al., 2011), while the probability to have multiple identical errors at a specific position at a given RAD locus is close to zero (Roesti et al., 2012). Overall, these risks imply that, for a de novo assembly, the minimum depth of coverage should be carefully chosen based on the error rate of the sequencing method, the read length, the assembly algorithms used and the repeat complexity of the genome studied (Schatz, Delcher, & Salzberg, 2010; Sims, Sudbery, Ilott, Heger, & Ponting, 2014). In practice, there are still few methods available for precise determination of the minimum depth of coverage required for such an analysis. Misalignments resulting from mapping errors due to repetitive regions or paralogous genes (for simplicity, both situations will be referred to as paralogous loci hereafter) are also likely to result in spurious identifications of loci as heterozygous (Bryc, Patterson, & Reich, 2013). Inversely, the clustering of one highly heterozygous locus into two loci can create false homozygotes (Ilut et al., 2014). Recently developed methods for the detection of paralogy in NGS data are based on the elimination of RAD loci containing too many SNPs or deviating from Hardy–Weinberg equilibrium (Lexer et al., 2014), the elimination of RAD loci with a too high coverage (Bianco et al., 2014), or on tests for the existence of two loci at each given position, as implemented in the paralogy filtering option of the reads2snp program (Gayral et al., 2013). These methods help to increase the efficiency of de novo assemblies of short reads and the detection of sequencing misalignments, resulting in more accurate SNP detection.

**Figure 1:**
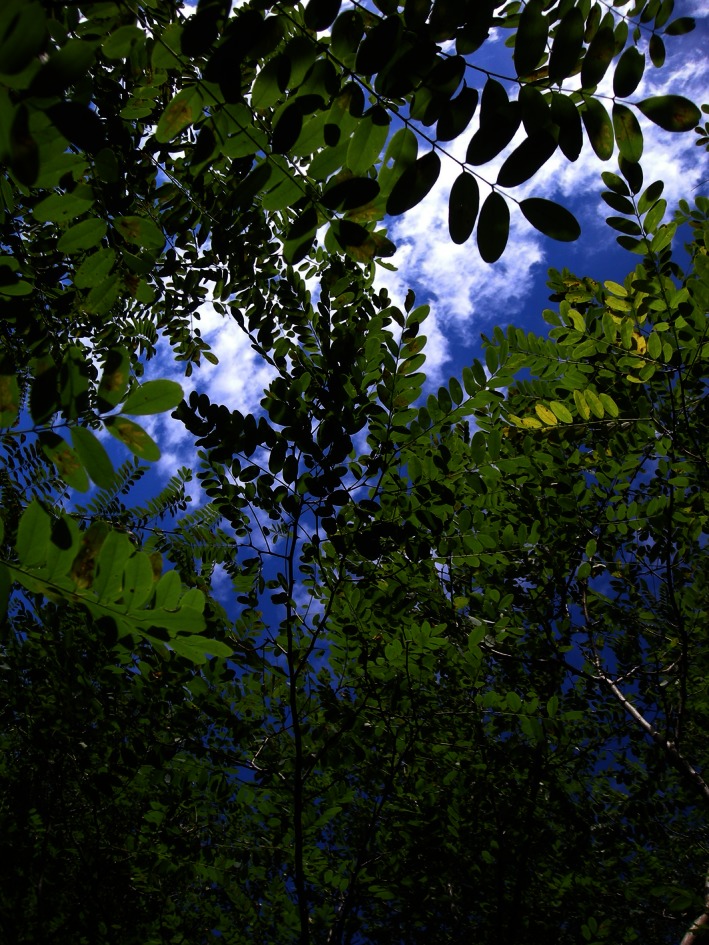
Photograph of *Robinia pseudoacacia* taken in Aquitaine (France)

*Robinia pseudoacacia* (Fig. 1, *Fabaceae* family) is native to the Eastern United States (Kennedy, 1983) and was introduced into Europe in the early 17th century (Cierjacks et al., 2013). Several parts of this tree have different uses (Barrett, Mebrathu, & Hanover, 1990), accounting for its widespread intentional introduction throughout temperate and subtropical regions of the world (Li, Xu, Guo, & Du, 2014). The species has efficiently spread subsequently and is now classified as invasive in many countries (Richardson & Rejmanek, 2011). This conflict creates the challenge to combine an increasing demand of forest managers to develop the cultivation of *R. pseudoacacia* in Europe with the limitation of its ecological impact by controlling its spread across the landscape. Appropriate new molecular markers are therefore required for *R. pseudoacacia*, both for initiating a breeding program and for studying the invasion dynamics of this species.

The first aim of this study was to develop SNPs for the nonmodel species *R. pseudoacacia*. The second aim was to show the importance of detecting and removing putative paralogy in RADseq data before performing population genetics inferences. Our approach included three steps: A first assembly of sequences to obtain a pseudo‐reference, a mapping of sequences using the pseudo‐reference, and a targeted detection of putative paralogy to eliminate polymorphism arising from paralogous sequences clustering. We added a validation step through genotyping to estimate the efficacy of the data cleaning with this approach.

## 2 Materials and Methods

### 2.1 Plant material

*Robinia pseudoacacia* seeds were collected from nine sites within the native (Ohio County, Monongalia County and Hardy County, West Virginia, USA) and non‐native (Pennsylvania, USA; New Jersey, USA; California, USA; Belgium; Italy and Iran) ranges (Table S1). Seeds were disinfected, scratched with sandpaper to break dormancy, and placed in a growth chamber for germination. One seedling per population was grown in a greenhouse for 3 months. Its leaves were then harvested for DNA extraction, sequencing, and SNP validation by genotyping.

### 2.2 Ploidy estimation

The ploidy of the sequenced plants was studied using two approaches. First, ploidy and DNA content were estimated for eight of the nine plants used (one individual died before the cytometrical analysis). Leaves or roots were chopped together with an internal standard (*Solanum lycopersicum* var. *cerasiformae* West Virginia 106) using a razor blade in a petri dish with 500 μl of Galbraith's nuclear‐isolation buffer, supplemented with 10 mmol/L sodium metabisulfite and 1% polyvinylpyrrolidone. The suspension was filtered through nylon mesh (pore size 50 μmol/L) and kept at 4°C. The nuclei were stained with 50 μg/ml propidium iodide after a 30‐min incubation with RNase A 10 U/ml. DNA contents of isolated nuclei were determined using a Partec CyFlow Space cytometer equipped with a 488‐nm laser and filter 630LP. The 2C DNA value was calculated using the linear relationship between the fluorescence signals from the first population of isolated stained nuclei of *R. pseudoacacia* studied species (*I*_Robinia_) and the known internal *S. lycopersicum* standard (*I*_Solanum_) according to the following equation: 2C_Robinia_ = *I*_Robinia_/*I*_Solanum_ × 2C_Solanum_.

Second, the ploidy of all nine samples was confirmed by analyzing ten microsatellites using the M13‐tail strategy (Schuelke, 2000): RP109, RP200, RP035, RP032, RP01B, RP106 from Mishima, Hirao, Urano, Watanabe, and Takata (2009) and ROPS05, ROPS06, ROPS08, ROPS10 from Lian and Hogetsu (2002). PCR products were diluted 400 times before separation on ABI‐3730 capillary sequencer (ThermoFisher Scientific).

### 2.3 RADseq experiment and quality filtering

Genomic DNA extraction and RAD library preparation were carried out in the laboratories of Ecogenics (Schlieren GmbH, Switzerland). Genomic DNA was extracted from dried leaves with the Qiagen (Venlo, the Netherlands) plant extraction kit following the manufacturer's protocol. Sequencing was carried out, and alignments were obtained with a double‐digest RAD approach. Genomic DNA was digested with *Eco*RI/*Mse*I and ligated to adapters suitable for Illumina sequencing. Individual libraries were tagged with the Trueseq i5 and i7 panel. The resulting reduced representation libraries were pooled, and size selection for fragments of 300 base pairs (bp)–400 bp was carried out by agarose gel electrophoresis and fragment extraction from the gel. Single‐end sequencing was performed on an Illumina v3 chip with the 1 × 150 bp format. The reduced representation libraries were around 2 Mb and the sequencing output per sample about 50 Mb, resulting in a mean depth of coverage of 20–30×, depending on sample considered.

All reads were trimmed to 100 bp. The quality of reads was then analyzed with the fastqc version 0.11.4 software (Andrews, 2015). Given the high quality of reads (per base sequence quality above 34 for all sequenced samples; see data accessibility section), no additional quality trimming was performed. However, residual Illumina adaptors were removed with the cutadapt version 1.10 software (Martin, 2011), and quality was checked again with fastqc.

### 2.4 Detection of putatively paralogous loci

Data were then analyzed with the program denovo_map.pl version 1.28 executing the stacks pipeline (Catchen, Amores, Hohenlohe, Cresko, & Postletwait, 2011). Default parameters were used except for the minimum depth of coverage required to create stacks (*m*) and the maximum distance in nucleotides allowed between stacks (*M*), see below for tested values.

**Figure 2:**
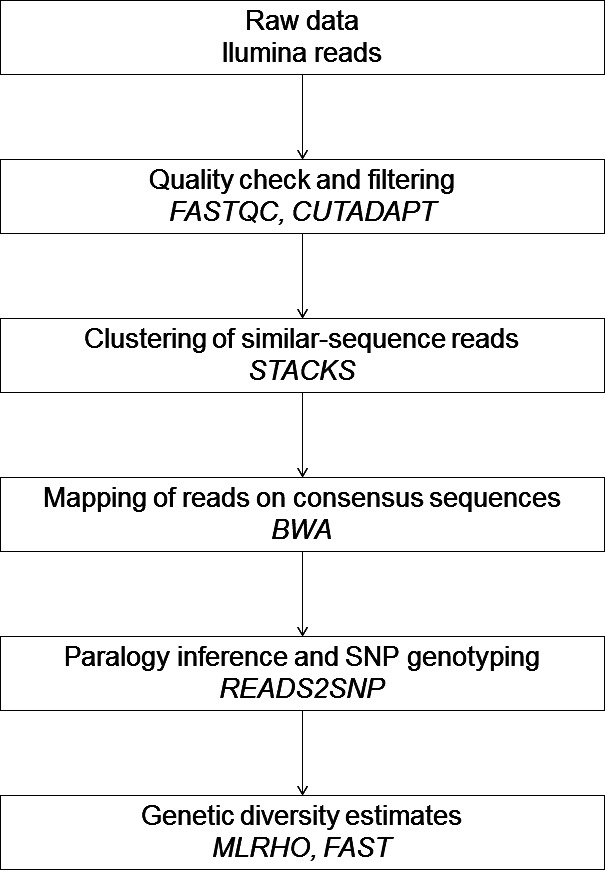
Outline of in silico data analyses

The consensus sequence of all the resulting identified RAD loci was used as a pseudo‐reference sequence in the subsequent analyses, to investigate the respective contributions of putative paralogy and depth of coverage to some population genetics estimates and SNP identification. Raw sequencing data were mapped onto these consensus sequences with bwa software version 0.7.12 (Li & Durbin, 2009), using the aln and samse options. The aligned reads were sorted and indexed using samtools version 1.2 (Li et al., 2009). The BAM files for each individual were then used both to identify putative paralogous loci and to obtain in silico candidate SNPs with reads2snp software version 1.0 (Gayral et al., 2013), using the paraclean option. Briefly, this method filters SNPs for potential paralogy with a likelihood ratio test. For each SNP position, the probability of the observed data under a one‐locus model and the probability of the observed data under a two‐locus model are compared. The two‐locus model makes the hypothesis that two paralogous loci account for the observed reads for the SNP, and it predicts an excess of heterozygotes. SNPs are validated if the two‐locus model does not improve the fit of the data. RAD loci were considered as paralogous if they contained at least one position annotated as “para” (suspicion of paralogy) with reads2snp software. They were discarded during the “without paralogs” analyses. The detection of paralogy with reads2snp was first tested with varying values of the minimum depth of coverage required to create stacks, *m* (from 2 to 20 with a step of two) and varying values of the maximum distance in nucleotides allowed between stacks, *M* (from 2 to 8 with a step of 2) for the stacks software. For all the following analyses, *M* was fixed to four (see Results). An outline of data analyses is presented in Fig. 2.

### 2.5 Comparison of the influences of putative paralogy and depth of coverage

#### 2.5.1 Sequencing error rate and diversity

Two methods were used to estimate the sequencing error rate and the nucleotide diversity at the entire sequence level. First, reads2snp was used to calculate the transition and transversion error rates for each RAD locus. The parameters were fixed as follows: “–min: 2–20, –th1: 0.95, –par: 1 and –th2: 0.01” where min is the minimal number of reads to call a genotype, th1 the genotype posterior probability threshold, par the paraclean option (1 = activated), and th2 the paraclean LRT *p*‐value threshold. The nucleotide diversity was assessed globally on the nine fasta format files (one for each sequenced individual) obtained with reads2snp using the alnpi program from the fast toolbox (Lawrence et al., 2015, https://github.com/tlawrence3/FAST).

The program mlrho was also used to estimate the global sequencing error rate and the nucleotide diversity for each individual separately (see Haubold, Pfaffelhuber, & Lynch, 2010 for the definitions of estimates; Lynch, 2008). The minimum (–*m*) and maximum (–*M*) length of reads were fixed at 1 and 100, respectively, with a step (–S) of one (Mariette et al., 2016).

The sequencing error rate and diversity were calculated with both methods, both with and without paralogous RAD loci, over an increasing range of minimal coverage depths (2×–20×). In addition, the mean coverage of RAD loci identified as paralogous or nonparalogous was estimated for each sequenced individual using samtools version 1.2 (Li et al., 2009) depth option. Format changes, to convert BAM files to input files for mlrho and reads2snp *,* were performed with custom‐designed python scripts developed in‐house (Python 2.7, https://www.python.org/).

#### 2.5.2 Detection of SNPs in silico

Single nucleotide polymorphisms were identified with reads2snp *,* with two contrasting minimal depths of coverage (4× and 18×), for both paralogous and nonparalogous RAD loci. For each depth of coverage, we calculated the numbers of monomorphic RAD loci, loci carrying one or two SNPs, and those carrying more than two SNPs, as well as the number of bi‐allelic and multiallelic SNPs and the mean number of SNPs per sequence both for paralogous and nonparalogous RAD loci, respectively. For paralogous RAD loci, we also estimated the number of loci containing at least one SNP not detected as putative paralog (called “pass” SNPs) and the proportion of “pass” SNPs among the total number of SNPs detected in these loci.

#### 2.5.3 Population genetics estimates

The impacts of putative paralogy filtering and minimal depth of coverage on diversity estimators were evaluated at the level of the individual SNPs. We used the genotypes of each detected SNP to estimate minor allele frequency (MAF), observed and expected heterozygosity (*H*_O_ and *H*_S_), and the inbreeding coefficient (*F*_IS_) following unbiased formula as found in Nei (1987, p. 164). Histograms were plotted, and the distributions of MAF and *F*_IS_ indices were compared using Wilcoxon signed‐rank tests in R version 3.2.1 (R Core Team 2015).

### 2.6 SNP genotyping

We used 10 ng of genomic DNA for genotyping with the iPLEX Gold genotyping kit (Sequenom) for the MassArray iPLEX genotyping assay (carried out according to the manufacturer's instructions). Products were detected in a MassArray mass spectrophotometer (Sequenom), and data were acquired in real time with MassArray RT software. All experiments were performed at the Bordeaux Genome Transcriptome Platform (INRA Pierroton, Cestas, France). Twelve multiplexes were designed for a total of 377 SNPs with MassArray assay design 4.1 software (Sequenom) and screened (Table S2). Focussing on RAD loci for which strictly more than six samples were genotyped in silico, the SNPs were designed from the consensus sequences given by the reads2snp software (171 SNPs detected at 4× only, 188 detected at 4× and 18×, 15 SNPs detected at 18× only, and three SNPs detected as paralogous SNPs). Clustering and genotype calling were performed automatically with MassArray TyperAnalyser 4.0.22 software, with the autocluster option, corrected, if necessary, by visual inspection. Each SNP was then classified on the basis of the number of clusters observed (A: three genotypic classes, two homozygotes and one heterozygote; B: two genotypic classes, one homozygote and one heterozygote; C: two genotypic classes, one for each homozygote; D: one homozygote genotypic class; E: one heterozygote genotypic class; F: unreadable SNPs; and G: unamplified SNPs). As the profile of class C could correspond to a plastid marker, we carried out a blast analysis with sequences containing these SNPs on the Chloroplast Genome Database (http://chloroplast.cbio.psu.edu/, Altschul et al., 1997).

The genotypes obtained in silico with reads2snp were compared with the genotypes obtained with MassArray using python scripts.

The genotyping data obtained with the MassArray assay for SNPs of classes A, B, and C, over the nine genotypes, were used for the calculation of MAF, *H*_O_, *H*_S_, and *F*_IS_ following unbiased formula as found in Nei (1987, p. 164). Comparison with results obtained in silico at both threshold, 4× and 18×, allowed us to estimate the impact of parameters chosen for cleaning data with reads2snp on diversity estimates.

## 3 Results

### 3.1 Ploidy estimation

The relative fluorescence (DNA content) obtained by flow cytometry is shown in Fig. S1. This histogram depicts a sharp peak and a low variance. The DNA peak ratio measured in the two different tissues (roots and leaves) of a single plant was constant. The eight cultivars of diploid *R. pseudoacacia* had similar 2C values ranging from 1.47 to 1.51 pg. No intraspecific variation was noticed. These results showed a uniform nuclear DNA content among the samples, consistent with the Plant DNA C‐values Database (Olszewska & Osiecka, 1984).

The microsatellite analysis confirmed the ploidy level of the nine individuals used for the sequencing (Table S1), as each sample showed either one or two alleles for each of the 10 markers tested.

### 3.2 Frequency of putative paralogy as a function of depth of coverage and mismatch between stacks

Both the total number of RAD loci and the putative paralogy detected with reads2snp were more dependent on the minimum depth of coverage than on the maximum distance in nucleotides allowed between stacks (Fig. S2). For example, the number of loci was more than 70 times higher at *m* = 2 than at *m* = 20 (*M* = 2), but only 1.05 times higher at *M* = 2 than at *M* = 8 (*m* = 2). Similarly, the percentage of paralogy was more than 20 times higher at *m* = 20 than at *m* = 2 (*M* = 2), but only 1.12 times higher at *M* = 8 than at *M* = 2 (*m* = 2). For the following analyses, *M* was consequently set at 4, at a level for which we hypothesized that loci are neither too oversplit nor too merged. The impact of the minimum depth of coverage was, however, investigated in detail.

### 3.3 Nucleotide diversity and sequencing error rate estimates as a function of putative paralogy and depth of coverage

Mean coverage was highly dependent on the minimal depth of coverage fixed for the analysis (Fig. S3). It was also always higher for paralogous RAD loci than for nonparalogous ones (3.5 times higher at depth = 2 and 2.5 times higher at depth = 20).

**Figure 3:**
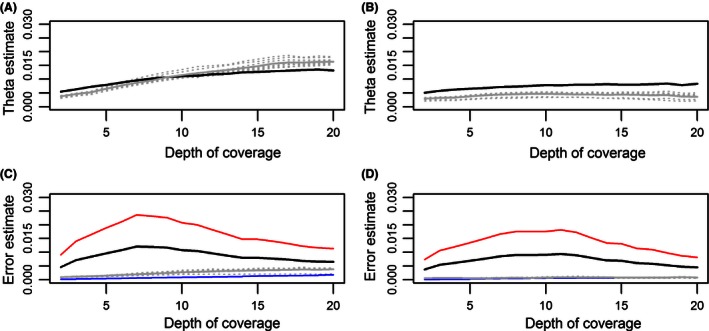
Effect of the depth of coverage on theta with (A) and without (B) paralogous RAD loci and on the error rate with (C) and without (D) paralogous RAD loci. Estimates obtained with the program mlrho are shown in gray, with dotted lines indicating the nine sequenced individuals and the solid line the mean value. Estimates obtained with fast (alnpi program) are shown in black (theta and error rate), red (transition error rate), and blue (transversion error rate)

Both estimated sequence diversity and error rates were sensitive to the actual presence of paralogous loci, and the overestimation due to putative paralogy was particularly detected at high depths of coverage (Fig. 3A, C to be compared with Fig. 3B, D, respectively). Both measurements were up to 4.5 times higher when estimated for all RAD loci than when estimated for nonparalogous RAD loci only. This overestimation was particularly marked for estimates made with mlrho. When removing the effect of putative paralogy (Fig. 3B, D), estimated sequence diversity and error rates also varied with the minimal depth of coverage, but to a lesser extent: The relative difference between the highest and lowest values of theta or the error rate was between 1.5 and 2.5 only, according to the software used. Finally, the change in sequence diversity with minimal depth of coverage also varied with paralogous filtering. If paralogous RAD loci were retained, then the theta value estimated with both programs increased with depth. If paralogous RAD loci were removed, theta slightly increased with depth until 10× and decreased thereafter if estimated with mlrho, whereas it increased with depth until 14× and then stabilized when estimated with alnpi.

### 3.4 Sequence polymorphism and in silico SNP detection as a function of putative paralogy and depth of coverage

Consistent with the results reported above, putative paralogy directly influenced the level of polymorphism measured at the sequence level: RAD loci identified as paralogous were more polymorphic than nonparalogous loci (Table 1). The number of SNPs per locus was higher for paralogous than for nonparalogous loci, and paralogous loci also contained a larger number of multiallelic SNPs.

**Table 1:** Results for single nucleotide polymorphism (SNP) detection with reads2snp software, considering RAD loci detected as paralogous (P) or nonparalogous (NP) for two minimal depths of coverage (4× and 18×)

	4×		18×	
	P	NP	P	NP
Number of RAD loci	3,451	85,341	873	3,453
Proportion of monomorphic RAD loci (%)	0	48.2	0	68.9
Proportion of polymorphic RAD loci with one or two SNPs (%)	16.4	31	20.4	20.3
Proportion of polymorphic RAD loci with more than two SNPs (%)	83.6	20.2	79.6	10.8
Number of SNPs	20,990	102,378	5,483	2,763
Mean number of SNPs/RAD locus	6.1	2.5	6.3	2.6
Proportion of bi‐allelic SNPs (%)	97.5	99.1	98.9	99.2
Proportion of tri/tetra‐allelic SNPs (%)	2.5	0.9	1.1	0.8
Number of paralogous RAD loci containing a “pass” SNP	2,983	–	793	–
Proportion of “pass” SNPs/total SNPs (%)	62.2	–	84.1	–

**Table 2:** Distribution of single nucleotide polymorphisms genotyped on the nine sequenced samples across the eight classes defined according to the number of clusters identified. See Materials and Methods for definitions of classes

	4× (%)	4× and 18× (%)	18× (%)
Two or three clusters (A, B, and C)	91.2	87.8	60.0
Monomorphic (D)	7.6	3.7	6.7
One heterozygote cluster (E)	0.6	0.5	0.0
Unreadable (F) or nonamplified (G)	0.6	8.0	33.3
Total	171	188	15

The results were coherent between both depths of coverage but, consistent with the results shown in Fig. S2, putative paralogy was much less frequent at 4× (4%) than at 18× (20%). At 4×, only 17% of SNPs were detected on paralogous RAD loci, whereas at 18×, this proportion reached 66.5%. However, paralogous loci may contain at least one SNP not detected as paralogous by reads2snp (called “pass” SNPs, Table 1). “Pass” SNPs represented up to 62.2% and 84.1% of the number of SNPs detected on paralogous loci at 4× and 18×, respectively (Table 1).

Higher levels of polymorphism were observed for 4× coverage than for 18× coverage (Table 1). At 4×, 48% of the RAD loci were monomorphic, 31% contained 1–2 SNPs, and 20% contained more than two SNPs. At 18×, 70% of the RAD loci were monomorphic, 20% contained 1–2 SNPs, and 11% contained more than two SNPs.

### 3.5 MAF and *F*_IS_ as a function of putative paralogy and depth of coverage

**Figure 4:**
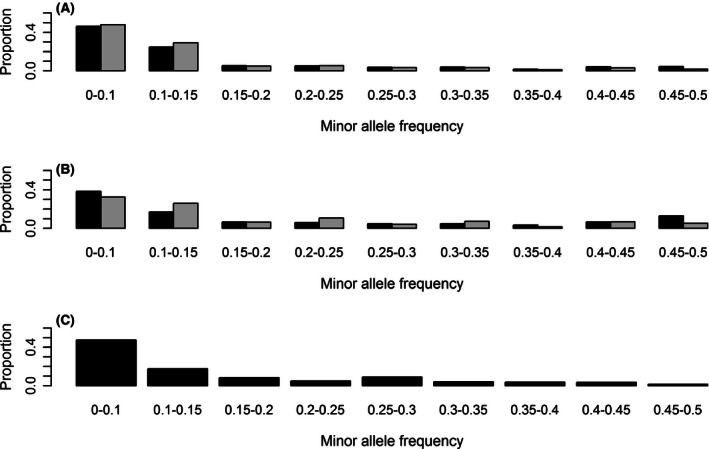
Distribution of minor allele frequencies (MAF) for RAD loci detected in silico with a minimum depth of coverage of 4× (A) and 18× (B), as well as for the 330 SNP loci validated by individual genotyping (C). For (A) and (B), the results obtained using all RAD loci are shown in black and those obtained after the removal of paralogous RAD loci are shown in gray

Figure 4A, B illustrates differences in the distribution of MAF between data including and excluding paralogous RAD loci, for each minimum depth of coverage: Reducing the information to loci with strictly more than four available in silico samples, 54,562 and 5,755 SNPs (including all RAD loci) and 36,886 and 991 SNPs (without paralogous RAD loci) were used for 4× (A) and 18× coverage (B), respectively. Contrasting results were obtained with and without paralogous RAD loci. At both coverages, an excess MAF of 0.45–0.5 was observed for all RAD loci with respect to the standard stationary distribution expected for a MAF distribution (Kim et al., 2011). This corresponds to an excess of RAD loci for which all samples were heterozygous. The removal of paralogous RAD loci decreased the relative number of SNPs with a MAF of 0.45–0.5 and increased either the relative number of SNPs with a MAF of 0–0.1, corresponding to rare alleles, or the relative number of SNPs with an intermediate MAF. The putative paralogy had a significant effect on the distributions, as revealed by significant Wilcoxon tests when comparing the distributions with and without paralogous loci at 4× (*p*‐value < 2.2e^−16^). The test was, however, not significant at 18×. The depth of coverage also had a significant effect on distributions (*p*‐values < 2.2e^−16^), when comparing the distributions at 4× and 18× for all loci, or when comparing the distributions at 4× and 18× excluding the paralogous loci.

**Figure 5:**
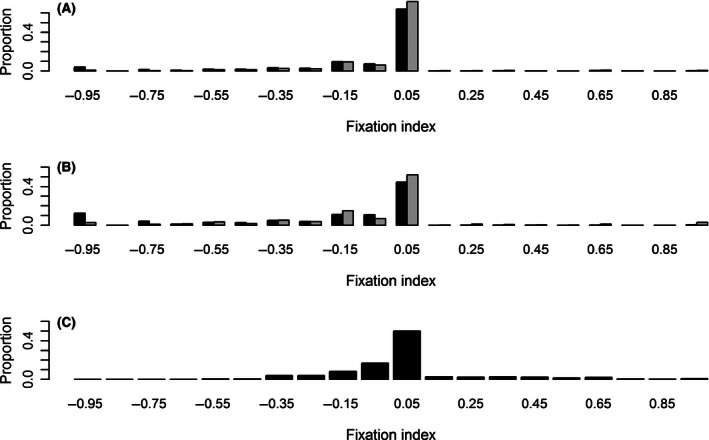
Distribution of inbreeding coefficients (*F*_IS_) for RAD loci detected in silico at 4× (A) and 18× coverage (B), as well as for the 330 SNP loci validated by individual genotyping (C). For (A) and (B), the results obtained using all RAD loci are shown in black and those obtained after the removal of paralogous RAD loci are shown in gray

The impact of the removal of paralogous RAD loci on the inbreeding coefficient (*F*_IS_) is shown in Fig. 5A, B. The removal of paralogous RAD loci increased the proportion of loci with *F*_IS_ values of 0–0.05 and decreased the one with a low *F*_IS_ value. The putative paralogy and the depth of coverage had both a significant effect on the *F*_IS_ distributions, as revealed by significant Wilcoxon tests when comparing the distributions with and without paralogous or when comparing the distributions obtained at 4× and 18× (*p*‐values < 2.2e^−16^).

### 3.6 SNP genotyping and impact of filters on the proportion of usable SNPs

In total, the validation of a given SNP was dependent on the depth of coverage at which it was discovered (Table 1 and Table S3 for detailed results). More than 90% of SNPs detected at 4× were validated, compared to 60% of SNPs detected at 18×. The nonvalidated SNPs were mostly monomorphic (up to 7%), unreadable, or nonamplified (less than 1% at 4× and more than 30% at 18×). Very few SNPs showed only a unique heterozygote cluster. In addition, none of the three SNPs detected as paralogous was validated as a true SNP in the genotyping, two of them being classified as monomorphic and only one showing a unique heterozygote cluster.

Overall, the genotyping validated 330 SNPs, which were assigned to class A, B, or C (sequences with SNP position are presented in Table S4). A third of these SNPs showed a highly unbalanced allele frequency with the rare variant present in only one genotype. Across the 330 validated SNPs, more than 90% of the genotypes obtained in silico were on average similar to those obtained on MassArray analysis (Table S5). The validation of the genotypes read at 4× for SNPs detected at 18× was a bit lower (83%), and the percentage of absent data (i.e., no genotype in silico) was higher when the 18× threshold was considered. The percentage of validation was somewhat lower for some combinations of individual and threshold.

The blast analysis carried out with the three sequences containing a class C SNP concluded that none of them resembled a chloroplast DNA sequence.

### 3.7 Estimation of MAF and *F*_IS_ from validated SNPs on MassArray

Minor allele frequency (Fig. 4C) and *F*_IS_ (Fig. 5C) were estimated for the nine sequenced individuals with the 330 validated SNPs classified A, B, or C. The distributions were very similar to those obtained in silico at 4× coverage without paralogous RAD loci (the *p*‐value of the Wilcoxon test was .003 for the MAF distribution and .087 for the *F*_IS_ distribution, respectively; all other comparisons gave highly significant tests).

## 4 Discussion

### 4.1 Putative paralogy biases population genetics estimates

RADseq technology is increasingly used in population genetics studies because it provides a rapid and cheap means for developing thousands of polymorphic SNP loci, almost regardless of genome size and previous genomic knowledge (Mastretta‐Yanes et al., 2015). However, we still know little about its potential biases and the consequences that errors in the analysis of sequencing data could have for genetic studies. In this study, we assessed how putative paralogy can bias population genetics estimates (at sequence or SNP level). Putative paralogy concerned a highly variable number of RAD loci depending on the depth of coverage, ranging from less than 1% of the RAD loci, when the depth was fixed to two, to more than 20% when the depth was fixed to 20. More than 60% of the SNPs detected in silico were located in RAD loci classified as paralogous, for the highest depths of coverage we tested. As we confirmed the ploidy level of the sequenced individuals to diploid, we assume that the rather high level of putative paralogy observed in this study comes from a high level of repeated sequences in the genome of *R. pseudoacacia*. This hypothesis is congruent with the ancestral whole genome duplication events which occurred within the *Fabaceae* (Cannon et al., 2014; Soltis et al., 2009). However, it may also be influenced by the fact that we considered all types of paralogous RAD loci, whereas we tested only considered those loci identified by reads2snp as true paralogs (“para”). Had we also included loci classified by the software as valid SNPs (“pass”), our estimate probably would have been somewhat lower. Nevertheless, this level of putative paralogy clearly exceeds previously reported values. Using the same approach, Gayral et al. (2013) inferred that 7%–37% of the detected SNPs were paralogous depending on the species considered. The lower rate of putative paralogy in this study may reflect the authors’ use of transcriptomes, which contain fewer repetitive sequences than ours.

All our analyses demonstrated a strong impact of paralogy on estimated levels of polymorphism (number of SNPs, number of multiallelic SNPs, theta, etc.), in line with but exceeding that of the depth of coverage. Consequently, neglecting paralogy when analyzing SNP data obtained with RADseq and mapped on a pseudo‐reference implies a major risk of bias in the estimates obtained (unless SNPs are validated by genotyping). Although less, diversity estimates were also influenced by the depth of coverage. Lower theta values were observed at very low depths of coverage, in regardless of the presence or absence of paralogous RAD loci. This is because a low depth of coverage may lead to missing minor alleles, which would result in an underestimation of diversity. Finally, the small decrease in theta with increasing depth, when theta is estimated with mlrho and when paralogous loci are removed, can be explained by a sampling effect, because, at high depth, fewer alleles are considered in the analysis, accounting for lower estimates of diversity.

Our analysis showed furthermore that putative paralogy and depth of coverage also influenced the estimation of the sequencing error rate. Both softwares (reads2snp and mlrho) provide sequence‐based estimations of error rates, in a maximum‐likelihood framework (Gayral et al., 2013; Haubold et al., 2010). It means that the estimation of the error is correlated with the polymorphism found in the sequence, and our results demonstrate this correlation and the relationship between the evolution of diversity and the evolution of the error rate with this type of method (compare Fig. 3A, B with Fig. 3C, D).

### 4.2 Impact of depth of coverage and putative paralogy on SNP validation

Besides reducing bias in diversity estimates on in silico RADseq data, the elimination of paralogous RAD loci can also help increase the success rate when screening SNPs for their quality. Thanks to our application of paralogy filtering for SNP identification, the genotyping revealed a very low percentage of SNPs with only a heterozygote group, this type of SNPs being putatively paralogous. Our genotyping of polymorphic positions identified as paralogous SNPs by reads2snp revealed that not a single one corresponded to a real SNP. They were either homozygous or heterozygous. Given the number of putative paralogous RAD loci observed, the cost of screening may be increased if no prior detection and filtering is performed. Interestingly enough, the larger number of SNP genotyping failure at 18× coverage than at 4× also suggests that the sequences provided by reads2snp at 18× may underestimate the polymorphism of the sequence, reducing the quality of primer design while increasing the number of mismatches during PCR and the number of unreadable or nonamplified SNPs.

Given the biases discussed above, we feel that a lower threshold is better: The total number of validated SNPs was higher at 4× (90%) than at 18× (60%).

### 4.3 Detecting paralogy in RADseq data

We used reads2snp to identify and exclude paralogous RAD loci. Consistent with our results, spurious SNPs due to putative paralogy can also be excluded by eliminating RAD loci with too many SNPs and markers deviating from Hardy–Weinberg equilibrium (Lexer et al., 2014). However, efficient strategies can be applied prior to population genetics analyses: Paired‐end sequencing can be used to infer loci from single original DNA fragments (Hohenlohe et al., 2013), linkage mapping can be used for identifying locus position, especially in highly duplicated genome species (Waples, Seeb, & Seeb, 2016). During the bioinformatics steps, Ilut et al. (2014) proposed a protocol to select the appropriate clustering threshold (*M*). Finally, paralogy should also be associated with overcoverage. In our study, the depth of coverage of paralogous RAD loci was roughly three times greater than that for nonparalogous. A strategy would therefore be to eliminate sequences with too high a coverage (Bianco et al., 2014).

## 5 Conclusion

In this study, we present a strategy for minimizing bias in RADseq analysis that allowed us to develop and validate 330 SNP markers for the nonmodel tree species *R. pseudoacacia*. Our validation by individual genotyping confirmed that the filtering of paralogous loci in silico with reads2snp software significantly increased the proportion of usable markers and the quality of data for population genomic studies. It also revealed that being too restrictive in the minimum depth of coverage during SNP screening loci can negatively affect the success rate of the validation procedure. The rate of SNP validation from RADseq studies for nonmodel species depends strongly on the species considered (e.g., 50%–77% for different conifer species, Karam et al., 2015). Because of the high validation rate, we can conclude that our strategy based on the elimination of paralogous RAD loci with reads2snp at a low threshold of coverage is a simple, efficient, and inexpensive way to improve the success rate of RADseq‐based SNP identification.

## Funding Information

Agence Nationale de la Recherche (Grant/Award Number: ANR‐10‐EQPX‐16 Xyloforest), Université de Liège (Grant/Award Number: ‘Special Research Fund 2014) and European Community's Seventh Framework Programme (EU FP7) (Grant/Award Number: Trees4Future 284181).

## Data Accessibility

Raw sequencing data and quality reports are available on DRYAD at doi:10.5061/dryad.qn4br.

## Conflict of Interest

None declared.
