# Supplementary figures and images for: Dealing with paralogy in RADseq data: in silico detection and single nucleotide polymorphism validation in Robinia pseudoacacia L
Source: Ecol Evol. 2016 Sep 22;6(20):7323–33. doi: 10.1002/ece3.2466 (PMC5513258; doi:10.1002/ece3.2466)

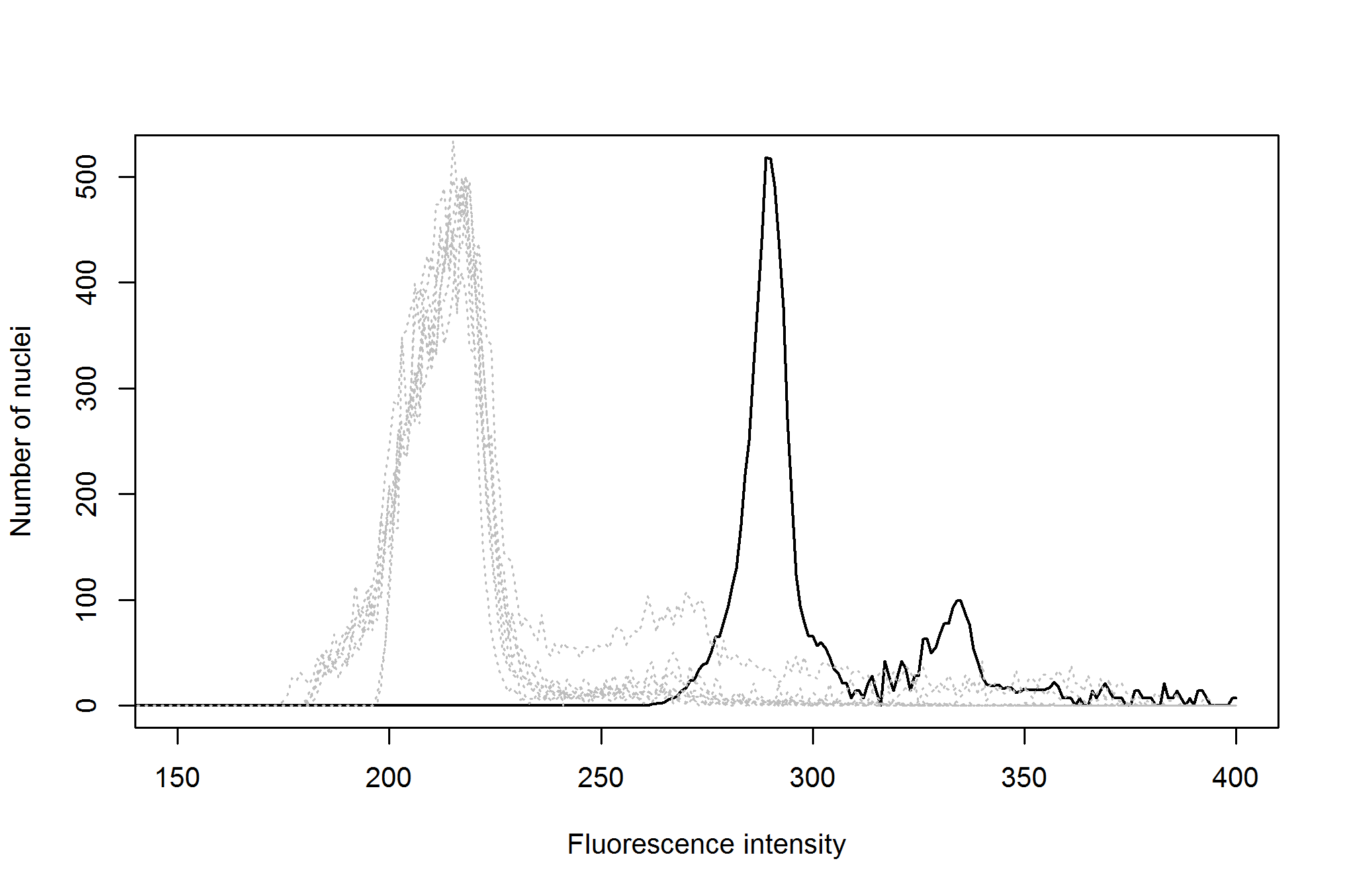

Supplement: Supplementary file 1 [file ECE3-6-7323-s001.tiff]

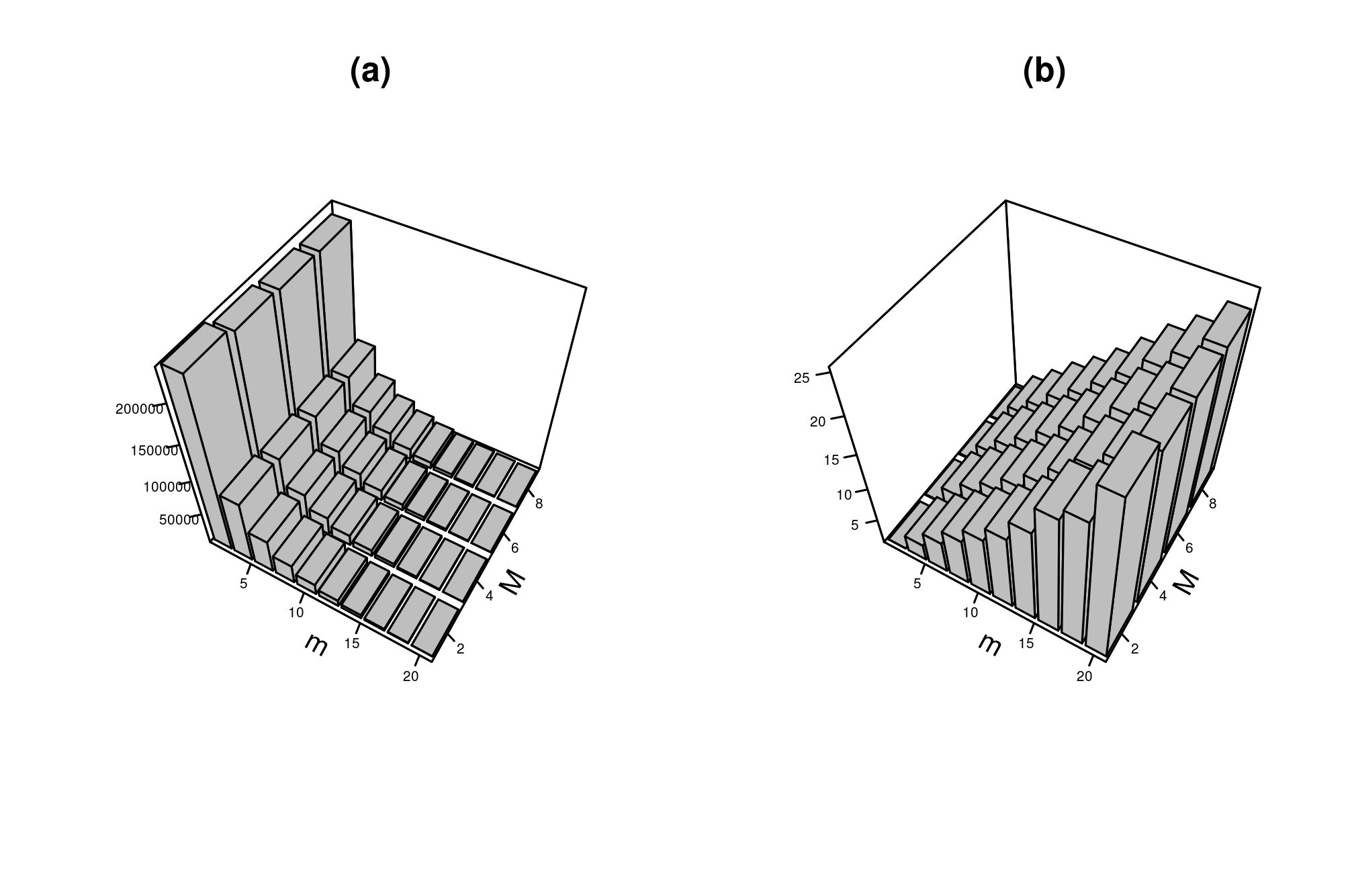

Supplement: Supplementary file 2 [file ECE3-6-7323-s002.tiff]

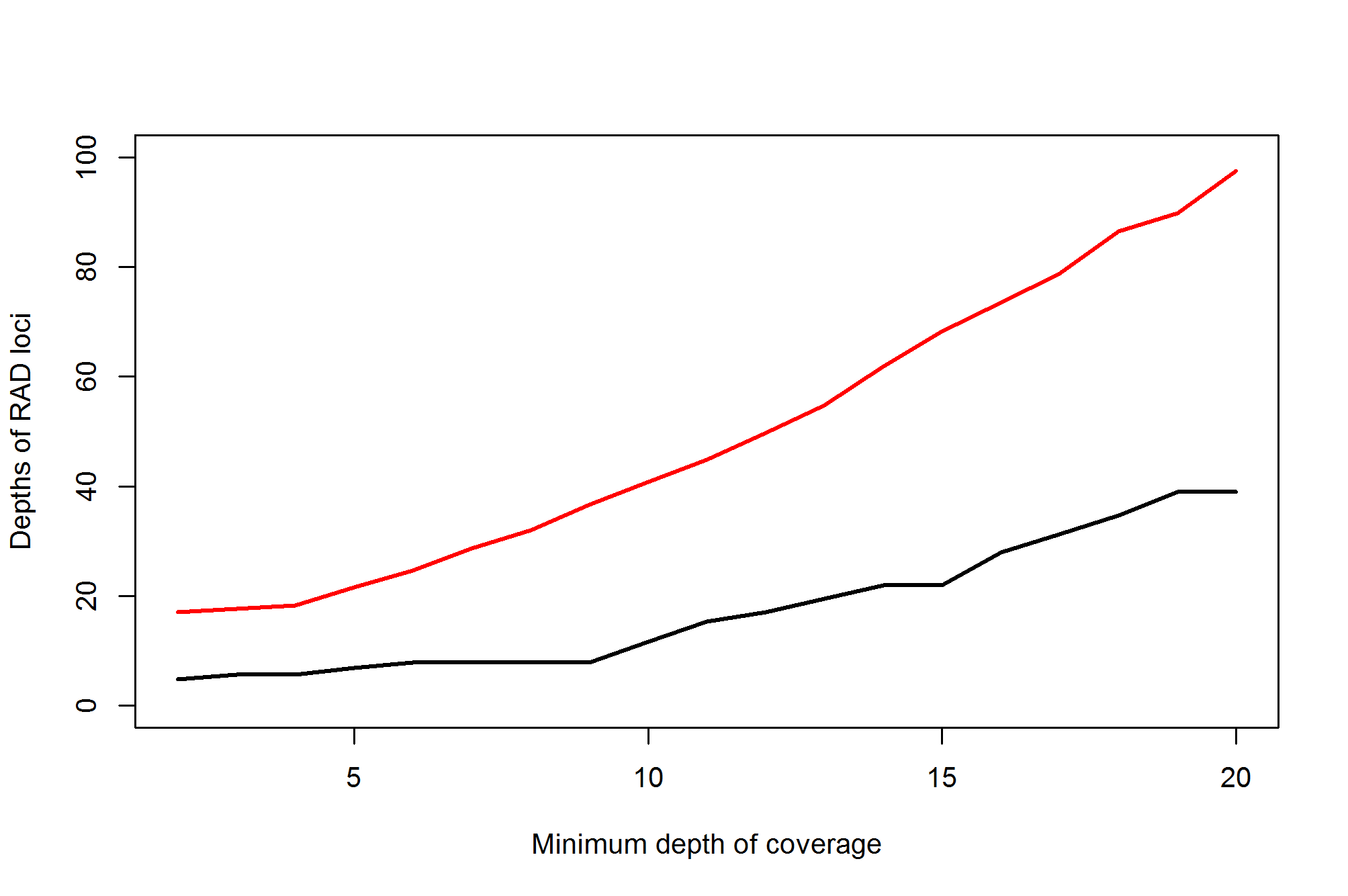

Supplement: Supplementary file 3 [file ECE3-6-7323-s003.tiff]
